# Direct comparison of two extended-half-life recombinant FVIII products: a randomized, crossover pharmacokinetic study in patients with severe hemophilia A

**DOI:** 10.1007/s00277-019-03747-2

**Published:** 2019-06-24

**Authors:** Anita Shah, Alexander Solms, Sara Wiegmann, Maurice Ahsman, Erik Berntorp, Andreas Tiede, Alfonso Iorio, Maria Elisa Mancuso, Tihomir Zhivkov, Toshko Lissitchkov

**Affiliations:** 1 Bayer, Whippany, USA; 2 Bayer, Berlin, Germany; 3 Bayer, Wuppertal, Germany; 4 LAP&P Consultants BV, Leiden, the Netherlands; 5 Centre for Thrombosis and Haemostasis, Lund University, Skåne University Hospital, Malmö, Sweden; 6 Department of Hematology, Hemostasis, Oncology and Stem Cell Transplantation, Hannover Medical School, Hannover, Germany; 7 Department of Health Research Methods, Evidence and Impact, and Department of Medicine, McMaster University, Hamilton, Canada; 8 Fondazione IRCCS Ca’ Granda, Ospedale Maggiore Policlinico, Angelo Bianchi Bonomi Hemophilia and Thrombosis Center, Milan, Italy; 9 Specialized Hospital for Active Treatment, Sofia, Bulgaria

**Keywords:** Pharmacokinetics, Extended half-life, Hemophilia A, PEGylated, Head-to-head study, Population pharmacokinetics

## Abstract

BAY 94-9027 is an extended-half-life, recombinant factor VIII (rFVIII) product conjugated with a 60-kDa branched polyethylene glycol (PEG) molecule indicated for use in previously treated patients (aged ≥ 12 years) with hemophilia A. This randomized, open-label, two-way crossover study compared the pharmacokinetics (PK) of BAY 94-9027 and rFVIII Fc fusion protein (rFVIIIFc) in patients with hemophilia A. Patients aged 18–65 years with FVIII < 1% and ≥ 150 exposure days to FVIII were randomized to receive intravenous single-dose BAY 94-9027 60 IU/kg followed by rFVIIIFc 60 IU/kg or vice versa, with ≥ 7-day wash-out between doses. FVIII activity was measured by one-stage assay. PK parameters, including area under the curve from time 0 to the last data point (AUC_last_, primary parameter), half-life, and clearance were calculated. Eighteen patients were randomized and treated. No adverse events were observed. In the analysis set excluding one outlier, geometric mean (coefficient of variation [%CV, 95% confidence interval {CI}]) AUC_last_ was significantly higher for BAY 94-9027 versus rFVIIIFc (2940 [37.8, 2440–3550] IU h/dL versus 2360 [31.8, 2010–2770] IU h/dL, *p* = 0.0001). A population PK model was developed to simulate time to reach FVIII threshold levels; median time to 1 IU/dL was approximately 13 h longer for BAY 94-9027 versus rFVIIIFc after a single infusion of 60 IU/kg. In conclusion, BAY 94-9027 had a superior PK profile versus rFVIIIFc. ClinicalTrials.gov: NCT03364998.

**Electronic supplementary material:**

The online version of this article (10.1007/s00277-019-03747-2) contains supplementary material, which is available to authorized users.

## Introduction

Prophylaxis with factor VIII (FVIII) is the standard treatment for patients with severe hemophilia A (FVIII < 1%) [1]. It aims to reduce bleeding frequency and, ultimately, prevent the development of chronic arthropathy [2–4]. However, prophylaxis regimens typically require frequent intravenous infusions, which can lead to suboptimal adherence and breakthrough bleeding [5]. Although the appropriate level of FVIII to prevent bleeding in individual patients varies depending on the individual’s pharmacokinetics (PK), bleeding phenotype, activity level, and other variables [6–8], an increased time with low FVIII levels is considered an important determinant of breakthrough bleeding during prophylaxis [9].

Extended-half-life (EHL) recombinant FVIII (rFVIII) products with improved PK profiles compared with standard-half-life (SHL) products have the potential to maintain FVIII levels above threshold levels for longer periods of time, which may result in better bleed protection and, consequently, less joint damage [10]. PK parameters, including incremental recovery, half-life (t_½_), area under the curve (AUC), and clearance (CL) are considered important surrogate efficacy endpoints for new FVIII products [11, 12]. EHL rFVIII products should have a minimum t_½_ extension ratio of 1.3 to provide a reduction in dosing frequency from three times per week to two times per week compared with SHL rFVIII products while maintaining the same minimum FVIII threshold level [13]. Such prophylaxis regimens that allow for less frequent infusions may also improve adherence [14].

BAY 94-9027 (Jivi®, Bayer AG, Germany) is a B-domain-deleted rFVIII product that has been site-specifically PEGylated with a single 60-kDa (dual-branched) polyethylene glycol (PEG) molecule to improve its PK [15]. In previously treated adults with severe hemophilia A, BAY 94-9027 demonstrated a longer t_½_ and greater dose-normalized area under the curve from time 0 to infinity (AUC_norm_) compared with sucrose-formulated rFVIII (Online Resource: Supplementary Table 1) [16, 17]. Subsequently, in the PROTECT VIII study and its extension, BAY 94-9027 was efficacious in the prevention of bleeds in previously treated adults and adolescents [18, 19]. These positive results led to the approval of BAY 94-9027 by the U.S. Food and Drug Administration (FDA), the European Medicines Agency (EMA) and the Pharmaceuticals and Medical Devices Agency (PMDA) in Japan for use in previously treated adults and adolescents (aged ≥ 12 years) with hemophilia A at dosing intervals of up to every 5 days (FDA) and every 7 days (EMA and PMDA) [20–22]. Population PK (popPK) evaluation of FVIII activity–time profiles following BAY 94-9027 dosing have shown that the PK of BAY 94-9027 is adequately described by a one-compartment model with linear elimination [23].

Recombinant FVIII Fc fusion protein (rFVIIIFc; Elocta®/Eloctate®; Biogen, Cambridge, MA, USA) is another EHL rFVIII product approved for routine prophylaxis for all age groups with dosing intervals of up to every 5 days [24]. In the A-LONG study, rFVIIIFc demonstrated a longer t_½_ and AUC_norm_ compared with conventional rFVIII (Advate®; Baxter, Deerfield, IL, USA) in previously treated patients aged ≥ 12 years with severe hemophilia A (Online Resource: Supplementary Table 1) [25]. The safety and efficacy of recombinant FVIIIFc has also been demonstrated for the prevention and treatment of bleeding episodes in studies of patients with severe hemophilia A [25, 26]. A two-compartment model with linear elimination has been reported to adequately describe the popPK of rFVIIIFc [27].

To date, no head-to-head comparison of the PK of EHL rFVIII products in patients with hemophilia A has been performed. The objective of the current study was to directly compare the PK profiles of BAY 94-9027 and rFVIIIFc. Concentration data collected using the one-stage assay were used to develop a popPK model for BAY 94-9027 and rFVIIIFc to simulate time to reach FVIII threshold levels.

## Methods

### Study design

**Fig. 1:**
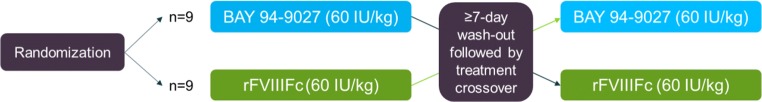
Study design

This was a single-center, randomized, open-label, single-dose, two-way crossover study (ClinicalTrials.gov identifier: NCT03364998) (Fig. 1). The primary objective was to compare the PK of BAY 94-9027 and rFVIIIFc. After a wash-out period (specified as ≥ 3 days or ≥ 5 days for SHL or EHL FVIII products, respectively), patients were randomized 1:1 to receive a single infusion of 60 IU/kg BAY 94-9027 or 60 IU/kg rFVIIIFc, followed by crossover to a single infusion of the other treatment, with ≥ 7-day wash-out between doses. The maximum wash-out time between treatments was 28 days. Both products were administered as 10-min intravenous infusions.

Vial strength was not determined in this study. One batch was used for each study drug. Study drug doses were based on the nominal value on the label of the vial. The exact volume needed for the administration of 60 IU/kg was calculated by multiplying the weight of the patient by 60. This total amount (IU) was withdrawn in a single pooling syringe using the required number of vials. The excess vial content was discarded to ensure that all subjects received a 60 IU/kg dose.

The study was approved by the institutional review board at the single site and was carried out in compliance with the protocol, the principles of the Declaration of Helsinki, and Good Clinical Practice guidelines. All patients gave written informed consent before initiation of any study-related procedures.

### Patients

Eligible patients were men aged 18–65 years with severe hemophilia A (FVIII <1 IU/dL) previously treated with any FVIII product for ≥ 150 exposure days (EDs). Patients also had to have a body mass index of 18–29.9 kg/m^2^ and have been able to stop FVIII treatment to complete the wash-out period before study entry and between treatments. Key exclusion criteria included the presence or history of an FVIII inhibitor (≥ 0.6 Bethesda units/mL), diagnosis of any bleeding disorder other than hemophilia A, platelet count < 75,000/mm^3^, HIV positive with a CD4 count of < 200/mm^3^, creatinine > 2 times the upper limit of normal (ULN) or alanine aminotransferase or aspartate aminotransferase > 5 times the ULN.

### PK assessments

Plasma samples were collected pre-dose and 0.25, 0.5, 1, 3, 6, 8, 24, 48, 72, 96, and 120 h after infusion of each drug. FVIII coagulant activity (FVIII:C) was measured using the same one-stage clotting assay as follows. Plasma concentrations of BAY 94-9027 and rFVIIIFc were determined by a turbidimetric assay with the SynthaSil reagent and activated partial thromboplastin time (APTT) measured on the ACL Advance System against a calibration curve of standard human plasma. The calibration range of the procedure for both BAY 94-9027 and rFVIIIFc was 1 IU/dL (lower limit of quantitation [LLOQ]) to 80 IU/dL (upper limit of quantitation [ULOQ]). Samples above the calibration range were diluted with FVIII-deficient plasma from human donors with congenital FVIII deficiency.

The following PK parameters were assessed using non-compartmental analysis (NCA) (WinNonlin® software, version 5.3; Pharsight, Mountain View, CA, USA): AUC from time 0 to the last data point (AUC_last_; primary parameter); AUC; maximum concentration (C_max_); t_½_; CL; mean residence time (MRT); volume of distribution at steady state (V_ss_); and incremental recovery.

### Population PK model

To evaluate differences in the PK of both EHL products in the specific study population, a single integrated PopPK model for BAY 94-9027 and rFVIIIFc was developed with product as the covariate. The analysis was conducted using the nonlinear mixed-effect modeling approach, as implemented in NONMEM® (version 7.4.1; ICON, Hanover, MD, USA). As a starting point, a structural model for each product was selected based on standard diagnostic tools, such as raw-data inspection, goodness of fit, and precision of parameter estimates. Potential candidates as suggested by previous analysis were one- or two-compartment models parameterized in terms of CL, central volume (Vc) and, for the two-compartment model, peripheral volume (Vp) and intercompartmental clearance (Q), with covariate effects of von Willebrand factor (VWF) and lean body weight (LBW) on CL and LBW on Vc. Residual (unexplained) variability was described using a combined (proportional and additive) error model. Data below the LLOQ were accounted for using the M3 method [28]. In the next step, an integrated model was developed by combining the two structural models and subsequently refining the model by testing whether BAY 94-9027 and rFVIIIFc have statistically significant differences in PK parameters (e.g., CL) using the likelihood ratio test (LRT) and a *p* value of 0.01. Because of the small study size, no additional covariate search was conducted. Additional model refinement consisted of an iterative outlier removal procedure and optimization of the inter-individual variability components of the model. The model was qualified using standard model diagnostic tools, such as uncertainty in parameter estimates, plausibility of estimates (comparison with published information), goodness-of-fit plots, and visual predictive checks.

The popPK model was used to determine individual PK estimates and simulate the time to reach FVIII threshold levels of 1, 3, 5, and 10 IU/dL after a single dose of 60 IU/kg BAY 94-9027 or rFVIIIFc for the study population.

### Safety

Safety was assessed by means of clinical and laboratory evaluation at study visits and the recording of adverse events.

### Statistical analysis

For statistical analysis of the PK parameters obtained by NCA, a log-normal distribution of the parameters was assumed [29]. Log-transformed parameters were analyzed using analysis of variance (ANOVA), including sequence, patient (sequence), period, and treatment effects. Based on these analyses, point estimates (least square means) and confidence intervals (CIs, 90% and 95%) for the BAY 94-9027:rFVIIIFc ratio were calculated by retransformation of the logarithmic data using intra-individual SD of the ANOVA. The lower limit of the 90% CI for the ratio exceeding 0.8 would indicate that BAY 94–9027 is non-inferior to rFVIIIFc; the lower limit of the 95% CI for the ratio exceeding 1.0 would indicate that BAY 94-9027 is superior to rFVIIIFc. Safety analyses were descriptive.

## Results

A total of 18 patients were randomized and received single doses of BAY 94-9027 and rFVIIIFc; the demographics and baseline characteristics of the patients are provided in Table 1. The mean age of patients was 36.0 years, all were white, and none had previously received EHL products.

**Table 1:** Patient demographics and baseline characteristics

Characteristic	Analysis set A (*N* = 18)	Analysis set B (*N* = 17)
Age, years		
Median (range)	34 (22–65)	34 (22–65)
Mean (SD)	36.0 (11.7)	36.1 (12.1)
Race, *n* (%)		
White	18 (100)	17 (100)
BMI, kg/m^2^		
Median (range)	25.5 (18.6–29.7)	25.0 (18.6–29.7)
Mean (SD)	24.8 (3.7)	24.7 (3.8)

*BMI*, body mass index; *SD*, standard deviation

Using data from all 18 patients (analysis set A), the geometric mean (%CV) for AUC_last_ was 2660 (60.6) IU h/dL for BAY 94-9027 and 2410 (32.1) IU h/dL for rFVIIIFc. The least square mean (90% CIs) for the BAY 94-9027:rFVIIIFc ratio was 1.10 (0.88–1.39), meeting the prespecified criteria for non-inferiority of BAY 94-9027 versus rFVIIIFc; superiority criteria were not met (95% CI 0.84–1.46; *p* = 0.46). Fifteen patients had a least square mean BAY 94-9027:rFVIIIFc ratio of > 1.0.

**Fig. 2:**
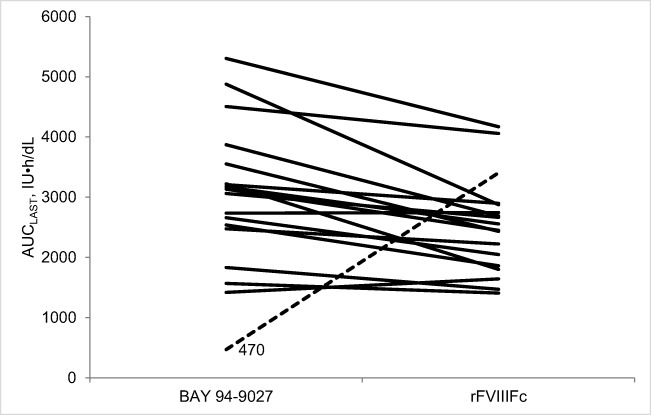
Individual patient AUC_last_ values after a single infusion of 60 IU/kg BAY 94-9027 or 60 IU/kg rFVIIIFc (*N* = 18). One patient (dashed line) had an AUC_last_ of 470 IU h/dL for BAY 94-9027, considerably lower than the geometric mean of 2660 IU h/dL for BAY 94-9027 for all patients

Examination of the individual patient AUC_last_ values after a single infusion of 60 IU/kg BAY 94-9027 or 60 IU/kg rFVIIIFc (Fig. 2), however, showed that one 34-year-old patient had an AUC_last_ of 470 IU h/dL for BAY 94-9027, considerably lower than the geometric mean of 2660 IU h/dL for BAY 94-9027 for all patients. This patient was the only one in the study to have pre-existing anti-PEG IgM (low titer 1:8) prior to administration of BAY 94-9027. For these reasons, this patient was determined to be an outlier and was therefore excluded from further analyses of the PK results (performed on the remaining 17 patients [analysis set B]).

Using analysis set B, the geometric mean (%CV, 95% CI) for AUC_last_ was significantly higher for BAY 94-9027 (2940 [37.8, 2440–3550] IU h/dL) versus rFVIIIFc (2360 [31.8, 2010–2770] IU h/dL, *p* = 0.0001, Table 2). Similar results were obtained for AUC (Table 2). CL was significantly reduced for BAY 94-9027 versus rFVIIIFc (0.0200 [38.3, 0.0165–0.0241] dL/h/kg versus 0.0250 [32.2, 0.0213–0.0294] dL/h/kg, *p* = 0.0001, Table 2). The geometric mean [%CV, 95% CI] t_½_ was significantly longer for BAY 94-9027 versus rFVIIIFc (16.3 [34.1, 13.7–19.3] versus 15.2 [33.1, 12.9–17.9] h, *p* < 0.05, Table 2). Additional PK parameters are shown in Table 2.

**Table 2:** PK parameters following single-dose administrations of BAY 94-9027 and rFVIIIFc (analysis set B, excluding outlier; *N* = 17)

Parameter	Geometric mean (%CV) (95% CI)		Geometric least square mean ratio^a^ (95% CI)	*p* value
	BAY 94-9027	rFVIIIFc		
AUC (IU h/dL)	3010 (38.3) (2490–3640)	2400 (32.2) (2040–2820)	1.26 (1.14–1.38)	0.0001
AUC_last_ (IU h/dL)	2940 (37.8) (2440–3550)	2360 (31.8) (2010–2770)	1.25 (1.14–1.37)	0.0001
CL (dL/h/kg)	0.0200 (38.3) (0.0165–0.0241)	0.0250 (32.2) (0.0213–0.0294)	0.80 (0.72–0.87)	0.0001
C_max_ (IU/dL)	150 (26.0) (131–171)	194 (64.1) (143–262)	0.76 (0.60–0.97)	< 0.05
MRT_IV_ (h)	23.2 (35.3) (19.4–27.6)	19.9 (38.4) (16.4–24.1)	1.17 (1.08–1.26)	< 0.001
t_½_ (h)	16.3 (34.1) (13.7–19.3)	15.2 (33.1) (12.9–17.9)	1.07 (1.00–1.15)	< 0.05
V_SS_ (dL/kg)	0.462 (15.2) (0.428–0.500)	0.497 (22.5) (0.444–0.558)	0.93 (0.86–1.00)	0.06
Incremental recovery (kg/dL)	2.26 (16.5) (2.08–2.46)	3.09 (66.0) (2.27–4.20)	0.72 (0.55–0.94)	< 0.05

^a^Ratio of BAY 94-9027:rFVIIIFc

*AUC*, area under the curve from time 0 to infinity; *AUC*_*last*_, AUC from time 0 to the last data point; *CL*, clearance; *C*_*max*_, maximum concentration; *MRT*_*IV*_, mean residence time after intravenous injection; *t*_*½*_, half-life; *V*_*ss*_, volume of distribution at steady state

**Fig. 3:**
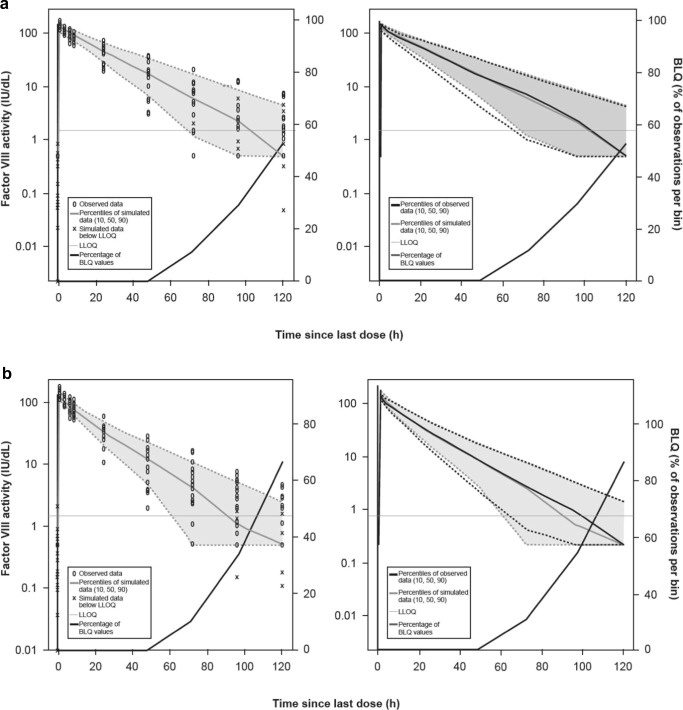
Visual predictive checks on FVIII level–time profiles in the integrated popPK model for BAY 94-9027 (**a**) and rFVIIIFc (**b**) *BLQ*, below the limit of quantification; *LLOQ*, lower limit of quantitation

The PK profile for BAY 94-9027 for the outlier patient was excluded from the development of the popPK model. No peripheral distribution compartment could be identified for BAY 94-9027 (relative standard error [RSE] of Q > 180%) and PK of BAY 94-9027 was described by a one-compartment model (technically, the PK of BAY 94-9027 was described by a two-compartment model fixing Q to a very small value [0.001]), while a two-compartment model was used for rFVIIIFc. Further, to minimize the potential bias introduced by implausible values (e.g., due to uncertainty of the assay or deviations in the sampling timepoint), single data points (ten measurements for BAY 94-9027 and 16 measurements for rFVIIIFc) were determined to be outliers and removed during model development. These single data points had a conditional weighted residual value (CWRES) of < −2.5 or > 2.5 (obtained using individual Bayesian post hoc parameter estimates) corresponding to a probability of occurrence under the respective model of < 1%. During this process, the estimate of the residual error was nearly halved to 29.7 %CV; this indicated that these data points were influential outliers and should be removed from the analysis. Compared with rFVIIIFc, the CL of BAY 94-9027 was significantly reduced by approximately 20% (95% CI, − 14.2 to − 26.9%). While all patients (excluding the outlier) had a lower CL for BAY 94-9027 compared with rFVIIIFc, the magnitude varied considerably between the subjects (%CV, 46%). The parameter estimates of the popPK model are shown in Table 3. Visual predictive checks showed good agreement between the popPK model and the observed data in that a statistically significant difference in CL could be detected between treatments (Fig. 3). The model parameters and results are consistent with previous popPK analyses [23, 27].

**Table 3:** Parameter estimates of the popPK model

Parameter	Value	RSE (%)	5% CI	95% CI
CL (dL/h)	1.57	10.5	1.25	1.89
Vc of distribution (dL)	28.3	3.59	26.3	30.3
Q (dL/h)^a^	0.69	20.2	0.42	0.96
Vp of distribution (dL)^a^	6.02	14.5	4.31	7.72
Effect of LBW on CL	1.03	32.9	0.364	1.69
Effect of LBW on Vc of distribution	1.10	15.5	0.765	1.43
Relative reduction of CL for BAY 94-9027 compared with rFVIIIFc^b^	− 0.21	14.0	− 0.26	− 0.15
Inter-individual variability in CL (variance [%CV])	0.11 (33.3)	28.5	0.05	0.16
Inter-individual variability in Vc of distribution (variance [%CV])	0.01 (11.1)	34.2	0.004	0.02
Inter-individual variability in change in CL for BAY 94-9027 compared with rFVIIIFc (variance [%CV])	0.20 (46.4)	51.4	− 0.002	0.39
Residual error, additive component (variance)	0.296	18.2	0.190	0.40
Residual error, proportional component (variance [%CV])	0.09 (29.7)	6.63	0.08	0.10

^a^Only applies for rFVIIIFc

^b^CL (BAY 94-9027) = CL (rFVIIIFc) × (1+ relative reduction in CL)

*CL*, clearance; *%CV*, coefficient of variation; *LBW*, low body weight; *Q*, intercompartmental CL; *RSE*, relative standard error; *Vc*, central volume; *Vp*, peripheral volume

**Fig. 4:**
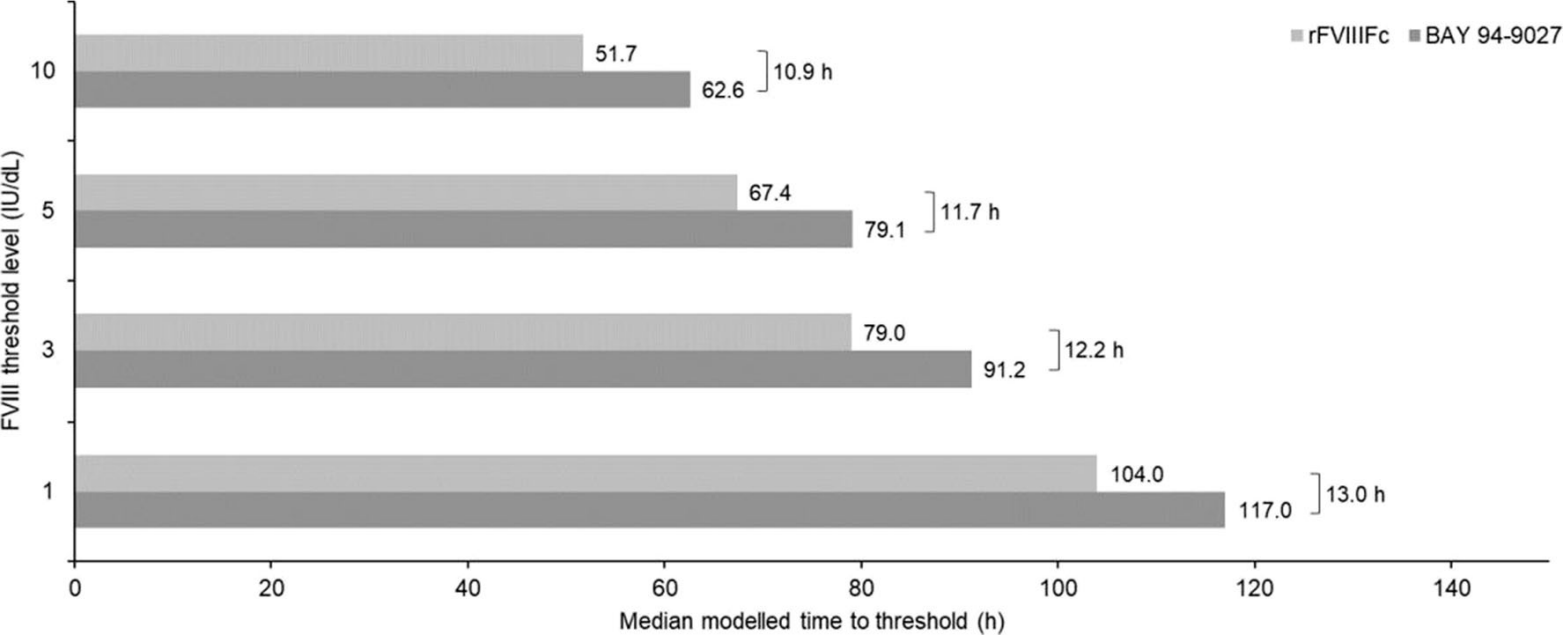
Modeled median time to FVIII threshold level after a single infusion of 60 IU/kg BAY 94-9027 or 60 IU/kg rFVIIIFc (analysis set B, excluding outlier; *N* = 17)

The popPK model was used to derive individual PK estimates and simulate time to reach FVIII threshold levels of 1, 3, 5, and 10 IU/dL after a single infusion of 60 IU/kg BAY 94-9027 or 60 IU/kg rFVIIIFc. For analysis set B (*N* = 17), median time to an FVIII level of 1 IU/dL was 13 h longer for BAY 94-9027 versus rFVIIIFc (approximately 12.5%). Times to reach 3, 5, and 10 IU/dL thresholds were 12.5, 11.7, and 10.9 h longer, respectively, for BAY 94-9027 versus rFVIIIFc (Fig. 4).

No adverse events were reported during the study.

## Discussion

This is the first randomized head-to-head study performed to directly compare the PK of BAY 94-9027 and rFVIIIFc following a single 60 IU/kg infusion in patients with hemophilia A. The results demonstrated that BAY 94-9027 has improved PK parameters compared with rFVIIIFc; the mean AUC_last_ was 25% higher and CL was 20% lower for BAY 94-9027 compared with rFVIIIFc.

The main strength of our study was the crossover design. Both products have previously been shown to have improved PK versus SHL rFVIII products [16, 17, 25]. The reported half-lives of the products based on registrational studies are 17.4 h for BAY 94-9027 [21] and 19.0 h for rFVIIIFc [30]. Supplementary Table 1 also describes PK parameters for these products based on published data. However, indirect comparisons of PK data from registrational studies do not allow for an accurate assessment of how the products compare owing to variation in the type of assay and calibration standard used and the characteristics of the patient populations. For example, one factor that influences PK is FVIII CL, which is highly inversely correlated with VWF levels in individual patients [31]. These issues reinforce the importance of our comparative crossover methodology, in which PK parameters were evaluated using the same assay in the same population of patients, allowing for direct comparison of the two products.

The clinical implication of our study is related to the concept that EHL rFVIII products can be used to extend the dosing interval [32] or provide higher FVIII levels for longer periods [33]. In this context, simulations using the popPK model showed that median time to a threshold level of 1 IU/dL FVIII was 13 h longer for BAY 94-9027 versus rFVIIIFc after a single infusion of 60 IU/kg. This increase in the time above threshold may thereby provide improved bleeding protection [9, 12]. However, only prospective studies can precisely assess the effects of improved PK on bleeding and individualized PK-based prophylaxis with BAY 94-9027.

One patient exhibited a lower AUC_last_ value for BAY 94-9027 than the other patients and was the only one found to have pre-existing anti-PEG IgM; he was therefore determined to be an outlier and was excluded from subsequent PK analyses. Pre-existing anti-PEG and anti-drug IgM have also been reported with BAX 855 and N8-GP, two other PEGylated FVIII products, and non-PEG therapeutics, such as biologic tumor necrosis factor (TNF) inhibitors [34–38]. Increased clearance, resulting in a reduced AUC_last_, of a drug secondary to pre-existing anti-PEG antibodies has been reported with other PEGylated therapeutics (e.g., PEG-asparaginase) [39].

Our study has some potential limitations. First, as a single chromogenic (two-stage) assay that could accurately measure FVIII activity of both BAY 94-9027 and FVIIIFc could not be identified, the same one-stage assay was used to assess FVIII activity for both products. The one-stage assay has been shown to give consistent results between PEGylated and non-PEGylated rFVIII [40], and it was found to accurately measure both products in the current study, with values within 20% for both products when analyzed against a plasma standard. However, the chromogenic assay measured values 40–60% higher than expected for rFVIIIFc and could not be validated. Therefore, the chromogenic assay was not used in the study. Second, NCA methods were used to compare the PK parameters, thereby providing a comparison that was unaffected by assumptions regarding the distribution of FVIII [41]. The popPK-model-based analysis, however, showed that a one-compartment model adequately described BAY 94-9027 but not rFVIIIFc, which was taken into account when simulating individual time-to-threshold values. Last, only patients aged 18–65 years were enrolled in this study. However, no major differences in the PK characteristics of BAY 94-9027 have been seen between adults and adolescents [17]. By contrast, the t_½_ of rFVIIIFc is decreased in adolescents aged 12–17 years compared with adults (aged ≥ 18 years) [42]. Taken together, these data suggest that the improved PK characteristics of BAY 94-9027 versus rFVIIIFc observed in adults in this study are likely to be seen also in adolescents.

In conclusion, BAY 94-9027 had an extended t_½_, a higher AUC (based on direct measurement), and longer median time to > 1 IU/dL FVIII (based on popPK modeling) compared with rFVIIIFc following a single infusion in patients with severe hemophilia A. Real-world data may provide an insight into whether these PK advantages provide additional bleeding protection.

## Electronic supplementary material

ESM 1 (DOCX 61 kb)

## Data Availability

Availability of the data underlying this publication will be determined according to Bayer’s commitment to the EFPIA/PhRMA “Principles for responsible clinical trial data sharing.” This pertains to scope, time point and process of data access. As such, Bayer commits to sharing upon request from qualified scientific and medical researchers patient-level clinical trial data, study-level clinical trial data, and protocols from clinical trials in patients for medicines and indications approved in the United States (US) and European Union (EU) as necessary for conducting legitimate research. This applies to data on new medicines and indications that have been approved by the EU and US regulatory agencies on or after January 01, 2014. Interested researchers can use www.clinicalstudydatarequest.com to request access to anonymized patient-level data and supporting documents from clinical studies to conduct further research that can help advance medical science or improve patient care. Information on the Bayer criteria for listing studies and other relevant information is provided in the study sponsors section of the portal. Data access will be granted to anonymized patient-level data, protocols, and clinical study reports after approval by an independent scientific review panel. Bayer is not involved in the decisions made by the independent review panel. Bayer will take all necessary measures to ensure that patient privacy is safeguarded.
