# Supplementary material for: Direct comparison of two extended-half-life recombinant FVIII products: a randomized, crossover pharmacokinetic study in patients with severe hemophilia A
Source: Ann Hematol. 2019 Jun 24;98(9):2035–44. doi: 10.1007/s00277-019-03747-2 (PMC6700041; doi:10.1007/s00277-019-03747-2)
Supplement: Supplementary file 1 — (DOCX 61 kb) [file 277_2019_3747_MOESM1_ESM.docx]

**Supplementary Table 1.** Comparison of PK parameters for EHL products (BAY 94-9027 and rFVIIIFc) versus standard rFVIII products

| **Products (reference)** | **t_½_, h** | | **AUC_norm_, kg•h/dL** | |
| --- | --- | --- | --- | --- |
|  | **EHL product** | **Comparator** | **EHL product** | **Comparator** |
| BAY 94-9027 versus rFVIII-FS [16,17] |  |  |  |  |
| Cohort 1* | 18.2 | 12.9 | 61.3 | 44.3 |
| Cohort 2^†^ | 18.5 | 13.0 | 72.1 | 50.0 |
| rFVIIIFc versus rFVIII^‡^ [25] | 19.0 | 12.4 | 51.2 | 32.9 |

*Sucrose-formulated rFVIII (rFVIII-FS) 25 IU/kg then BAY 94-9027 25 IU/kg twice weekly for 8 weeks. ^†^rFVIII-FS 50 IU/kg then BAY 94-9027 60 IU/kg once weekly for 8 weeks. ^‡^Advate^®^; Baxter, Deerfield, IL, USA.
